# Icariin Protects Bone Marrow Mesenchymal Stem Cells Against Iron Overload Induced Dysfunction Through Mitochondrial Fusion and Fission, PI3K/AKT/mTOR and MAPK Pathways

**DOI:** 10.3389/fphar.2019.00163

**Published:** 2019-02-28

**Authors:** Xudong Yao, Xingzhi Jing, Jiachao Guo, Kai Sun, Yi Deng, Yong Zhang, Fengjing Guo, Yaping Ye

**Affiliations:** Department of Orthopedics, Tongji Hospital, Tongji Medical College, Huazhong University of Science and Technology, Wuhan, China

**Keywords:** iron overload, icariin, bone marrow mesenchymal stem cells, mitochondrial fusion and fission, PI3K/AKT/mTOR pathway, MAPK pathway

## Abstract

Iron overload has been reported to contribute to bone marrow mesenchymal stem cells (BMSCs) damage, but the precise mechanism still remains elusive. Icariin, a major bioactive monomer belonging to flavonoid glucosides isolated from Herba Epimedii, has been shown to protect cells from oxidative stress induced apoptosis. The aim of this study was to investigate whether icariin protected against iron overload induced dysfunction of BMSCs and its underlying mechanism. In this study, we found that iron overload induced by 100 μM ferric ammonium citrate (FAC) caused apoptosis of BMSCs, promoted cleaved caspase-3 and BAX protein expressions while inhibited Bcl-2 protein expression, which effects were significantly attenuated by icariin treatment. In addition, iron overload induced significant depolarization of mitochondrial membrane potential (MMP), reactive oxygen species (ROS) generation and inhibition of mitochondrial fusion/fission, which effects were also attenuated by icariin treatment. Meanwhile, we found that iron overload induced by 100 μM FAC significantly inhibited mitochondrial fission protein FIS1 and fusion protein MFN2 expressions, inhibited DRP1 and Cytochrome C protein translocation from the cytoplasm to mitochondria. Icariin at concentration of 1 μM was able to promote mitochondrial fission protein FIS1 and fusion protein MFN2 expressions, and increase DRP1 and cytochrome C protein translocation from the cytoplasm to mitochondria. Further, osteogenic differentiation and proliferation of BMSCs was significantly inhibited by iron overload, but icariin treatment rescued both osteogenic differentiation and proliferation of BMSCs. Further studies showed that icariin attenuated iron overload induced inactivation of the PI3K/AKT/mTOR pathway and activation of the ERK1/2 and JNK pathways. In summary, our study indicated that icariin was able to protect against iron overload induced dysfunction of BMSCs. These effects were potentially related to the modulation of mitochondrial fusion and fission, activation of the PI3K/AKT/mTOR pathway and inhibition of ERK1/2 and JNK pathways.

## Introduction

Bone marrow mesenchymal stem cells (BMSCs) are well known for the ability to self-renewal and a latent ability to differentiate into cell types such as osteoblasts, adipocytes, myoblasts, and chondrocytes (Caplan, 1991; Ye et al., 2012). BMSCs have also been shown to represent a useful progenitor cell source for both cell therapy and tissue engineering. Many studies have shown that BMSCs transplantation into sites such as myocardium and brain would have a beneficial effect on the function of target organs, although it is still controversial whether transplanted BMSCs could differentiate directly into theses specific cell types such as cardiomyocytes or neurons (Phinney and Prockop, 2007; Picinich et al., 2007). Investigations have also demonstrated that the therapeutic effects of BMSCs may be related to the ability of BMSCs homing to sites of inflammation or tissue injury (Penn et al., 2009). MSCs could secrete massive bioactive immune-modulatory molecules or trophic factors (Meirelles Lda et al., 2009).

Recently a randomized, triple-blind and placebo controlled phase1/2 clinical trials showed that intra-articular implantation of autologous BMSCs could be a promising treatment choice for knee osteoarthritis (Emadedin et al., 2018). Many investigations have also demonstrated that BMSCs have a great potential for bone repair and bone regeneration (Chiu et al., 2014; Sponer et al., 2018). Generally, the osteogenic differentiation of BMSCs is regulated by the well-known TGFβ/Smad and BMP signaling pathway, Wnt/β-catenin signaling pathway (Liu et al., 2014; Zhang et al., 2016). Some transcriptional factors such as Osterix and Runx2 have also been proven to play important roles in regulating osteogenic differentiation of BMSCs (da Silva et al., 2018). On the other side, adipogenic differentiation of BMSCs is regulated by the important transcriptional factors such as peroxisome proliferator-activated receptor γ (PPARγ) and CCAAT/enhancer-binding protein (C/EBP) (Li et al., 2014; Zhu et al., 2018). Maintaining a balance between osteogenesis and adipogenesis in BMSCs is critical for the bone homeostasis. BMSCs could also provide a supportive microenvironment for hematopoiesis and are related to the maintenance of the sinusoidal walls in the bone marrow (Sacchetti et al., 2007). Investigations have also shown that in osteoporosis there is an increase in the adipocytes content, BMSCs obtained from the osteoporotic postmenopausal women are characterized by increased adipogenic potential (Rodriguez et al., 2009).

Iron is essential for hemoglobin synthesis and many vital enzymatic functions, and iron balance is critical for the growth and survival of many tissues and organs (Yang et al., 2017). Iron deficiency results in anemia and sometimes organ dysfunction (Andrews, 2000). On the contrary, iron overload is characterized by excessive ion deposition in organs throughout the body including liver, heart, and endocrine glands (McDowell and Sticco, 2018). Primary iron overload disease is often inherited with hereditary hemochromatosis be the leading cases (Kawabata, 2018). Secondary iron overload is often caused by the introduction of excessive iron into the body such as through blood transfusion, hemolysis or excessive dietary consumptions (McLaren et al., 1983). Excessive iron deposition in the human body could lead to chronic liver cirrhosis, heart failure or irregular heart rhythms, infertility, arthritis and degenerative neurological disease (Cogswell et al., 1998). Age-associated iron accumulation is confirmed to be a novel pathogenic factor for postmenopausal osteoporosis (Liu et al., 2006). Besides, iron is also involved in the development of osteoporosis in patients characterized by iron overload such as hereditary hemochromatosis and β-thalassaemia (Valenti et al., 2009; Baldini et al., 2014). Iron overload also drastically injured the bone marrow microenvironment in mice and disturbed the proliferation potential and differentiation balance of BMSCs (Zhang et al., 2015). Iron overload could break the anti-oxidant and pro-oxidant balance in cells by reactive oxygen species (ROS) mediated reaction and causing damage to DNA, proteins and lipids (Zheng et al., 2017).

Icariin, a major bioactive monomer belonging to flavonoid glucosides isolated from Herba Epimedii, has been shown to present multiple effects such as regulating sex hormone, relieving atherosclerosis, treating immunological and inflammatory diseases and antioxidant activity (Shen and Wang, 2018). Recent investigations have also indicated that icariin treatment effectively attenuated bone loss in ovariectomy induced osteoporotic rats (Wang Q.S. et al., 2018). In addition, a 24-month randomized double-blind placebo-controlled clinical trial demonstrated that icariin was effective in preventing postmenopausal osteoporosis with relatively low side effects (Wang Z. et al., 2018). Icariin has also been shown to promote the osteogenic differentiation of BMSCs and adipose-derived mesenchymal stem cells (ASCs) (Wei Q. et al., 2016; Ye et al., 2017). Recent studies also revealed that icariin could protect endothelial progenitor cells from oxidative stress induced apoptosis and hypoxia induced osteoblast apoptosis (Ma et al., 2014; Tang et al., 2015).

However, whether icariin treatment can protect BMSCs against iron overload induced damage, as well as the underlying molecular mechanism remains elusive. Therefore the aim of the present investigation was to characterize the effects of icariin and to study whether icariin treatment could antagonize the iron overload induced dysfunction of BMSCs.

## Materials and Methods

### Reagents

Icariin (United States, 489-32-7) and ferric ammonium citrate (FAC, United States, 1185-57-5) were purchased from Sigma-Aldrich (Supplementary Figure S1). Cleaved caspase-3 (Asp175) antibody (United States, #9661), MFN2 antibody (United States, #11925), DRP1 antibody (United States, #8570), COX IV antibody (United States, #4850), RUNX-2 antibody (United States, #12556), active β-catenin antibody (United States, #19807), cyclin D1 antibody (United States, #2922), PI3K antibody (United States, #4257), P-PI3K antibody (United States, #4228), AkT antibody (United States, #4691), P-AkT antibody (United States, #4060), mTOR antibody (United States, #2972), P-mTOR antibody (United States, #5536), P-ERK antibody (United States, #4370), ERK antibody (United States, #4695), P-p38 antibody (United States, #4511), p38 antibody (United States, #8690), P-JNK antibody (United States, #4668), JNK antibody (United States, #9252) were all purchased from Cell Signaling Technology. BAX antibody (United States, 50599-2-Ig), FIS1 antibody (United States, 10956-1-AP), cytochrome C antibody (United States, 66264-1-Ig) and osteopontin (OPN) antibody (United States, 22952-1-AP) were all purchased from Proteintech (United States, 50599-2-Ig). Bcl-2 antibody was purchased from R&D system (United States, MAB8272). Anti-beta actin antibody was purchased from BOSTER (China, BM3873).

### Isolation and Culture of BMSCs

Two-weeks old Male Sprague-Dawley (SD) rats were purchased from the Laboratory Animal Center of the Tongji Medical College, Huazhong University of Science and Technology (Wuhan, China) and used in the our study. All the animal procedures were approved by the Institutional Animal Care and Use Committee of Tongji Medical College, Huazhong University of Science and Technology (IACUC number TJ-A20161208). Rat BMSCs were isolated, cultured, and characterized as described in previous study (Wang Y. et al., 2018). Following euthanasia, the femur and tibia of SD rats were obtained and bone marrow was flushed out with Dulbecco’s modified Eagle’s medium (DMEM)/F12 with 10% fetal bovine serum (FBS). Primary cells were cultured at 37°C in 5% humidified CO_2_. After 48 h, non-adherent cells were removed and culture medium was changed every 2 days. When reaching 80–90% confluence, cells were digested with 0.25% trypsin and then seeded to three culture flasks. Third passage of the isolated BMSCs was used for further experiments.

### Cell Viability Assay

A cell counting kit-8 (Boster, Wuhan, China) was used to measure cell viability. BMSCs were seeded in 96-well plates at a density of 1 × 10^4^ cells/well and treated with different concentrations of FAC (0, 10, 50, 100 μM) for 24 and 48 h, respectively. FAC was dissolved into DMEM/F12 medium with 10% FBS. Then 10 μl of CCK-8 solution was added to each well at 37°C for 4 h to form WST-8 formazan. OD values of each well were read and recorded at 450 nm with a microplate reader according to manufacturer’s instructions.

### Cell Apoptosis Evaluated by Annexin V-FITC/PI Staining

Bone marrow mesenchymal stem cells were initially seeded in 6-well plates at a density of 4 × 10^5^ cells/well. After 24 h, cells were treated by 100 μM FAC or 100 μM FAC combined with different concentrations of icariin (0.01, 0.1, 1, 10 μM) for 48 h. In brief, icariin was diluted to different concentrations to ensure that the concentration of DMSO is the same (0.1%) in each group. The control group was treated with 0.1% DMSO. After treatment, cells were washed with phosphate buffered saline (PBS) three times and harvested to centrifuge tubes. Staining was performed with Annexin V-FITC Apoptosis Detection Kit (C1063, Beyotime) for 15 min at room temperature in the dark according to manufacturer’s protocol. Annexin V^+^/PI^-^ and Annexin V^+^/PI^+^ cells were considered as early and late phase apoptotic cells. The results were analyzed with FACS Calibur flow cytometer (BD, Franklin Lakes, NJ, United States).

### Detection of Mitochondrial Membrane Potential

Bone marrow mesenchymal stem cells were initially seeded in 6-well plates at a density of 4 × 10^5^ cells/well. After 24 h, cells were treated by 100 μM FAC or 100 μM FAC combined with different concentrations of icariin (0.01, 0.1, 1, 10 μM) for 48 h. After treatment, the mitochondrial membrane potential (MMP) was detected with mitochondrial membrane potential assay kit with JC-1 (C2006, Beyotime, China) according to manufacturer’s instruction. In brief, cells were washed with PBS and stained with JC-1 staining solution for 20 min at room temperature in darkness. After washing with JC-1 buffer, cells were observed under the fluorescence microscope (Evos fl auto, Life Technologies, United States) and images were taken at a magnification of 200-fold. JC-1 exhibits potential dependent accumulation in mitochondria, mitochondria with normal membrane potential was accumulated with high concentration of JC-1 aggregates and produce red fluorescence. Under circumstances of cell apoptosis, the MMP collapsed and JC-1 failed to aggregate in the mitochondria. JC-1 then existed in the form of monomer and the mitochondria were labeled in green. After treatment, cells were also resuspended with culture medium and the ratio of red fluorescence intensity was measured with FACS Calibur flow cytometer (BD, Franklin Lakes, NJ, United States).

### Detection of Intracellular Reactive Oxygen Species (ROS)

Bone marrow mesenchymal stem cells were initially seeded in 6-well plates at a density of 4 × 10^5^ cells/well. After 24 h, cells were treated by 100 μM FAC or 100 μM FAC combined with different concentrations of icariin (0.01, 0.1, 1, 10 μM) for 48 h. After treatment, the intracellular ROS level was determined with the Reactive Oxygen Species Assay Kit (S0033, Beyotime, China) according to manufacturer’s instructions. The probe DCFH-DA could pass freely through cell membrane and oxidized by the intracellular ROS to form the highly fluorescent compound DCF. In brief, cells were washed with PBS and treated with 10 μM DCFH-DA for 20 min at 37°C in the dark. After incubation, cells were washed with PBS and observed under the fluorescence microscope (Evos fl auto, Life Technologies, United States) and images were taken at a magnification of 200-fold. To quantify ROS levels, cells were also collected, resuspended, and treated with 10 μM DCFH-DA for 20 min at 37°C in the dark. After incubation, cells were rinsed with serum-free DMEM and finally the mean fluorescence intensity (MFI) was measured with FACS Calibur flow cytometer (BD, Franklin Lakes, NJ, United States).

### Mitochondrial Specific Fluorescence Staining

Bone marrow mesenchymal stem cells were initially seeded in 6-well plates at a density of 4 × 10^5^ cells/well. After 24 h, cells were treated by 100 μM FAC or 100 μM FAC combined with 1 μM icariin for 48 h. After treatment, cells were washed with PBS twice and incubated with 20 nM Mito-Tracker Green (C1048, Beyotime, China) working solution for 30 min at 37°C in the dark. After incubation, cells were washed with cell culture medium and observed under the fluorescence microscope (Evos fl auto, Life Technologies, United States) and images were taken at a magnification of 400-fold and further zoomed digitally.

### Osteogenic Differentiation of BMSCs and Alizarin Red Staining

Bone marrow mesenchymal stem cells were seeded in 12-well plates at a density of 1 × 10^5^ cells/well and cultured in regular culture medium (DMEM with 10% FBS and 1% penicillin/streptomycin) until 80–90% confluence. Then the medium was replaced with DMEM containing 5 mM β-glycerophosphate, 10^-7^ M dexamethasone and 0.1 mM ascorbate-2-phosphate, or replaced with DMEM containing 5 mM β-glycerophosphate, 10^-7^ M dexamethasone, 0.1 mM ascorbate-2-phosphate and FAC (100 μM) or replaced with DMEM containing 5 mM β-glycerophosphate, 10^-7^ M dexamethasone, 0.1 mM ascorbate-2-phosphate, FAC (100 μM) combined with 1 μM icariin. After induction of osteogenic differentiation for 3 weeks, osteogenesis was evaluated with Alizarin Red (Cyagen Biosciences, Guangzhou, China) staining according to the manufacturer’s protocols and images were scanned with a 40-fold microscope for the gross Alizarin Red staining and further taken at a magnification of 100-fold.

### 5-Ethynyl-2′-Deoxyuridine (EdU) Staining

Proliferation of BMSCs was investigated with BeyoClick^TM^ EdU Cell Proliferation Kit (C0075S, Beyotime, China) according to the manufacturer’s protocols. In brief, proliferating BMSCs were washed with PBS twice and incubated with Edu working solution (10 μM) for 4 h at 37°C in the dark. After incubation, BMSCs were washed with PBS twice and fixed with 4% paraformaldehyde for 15 min. Next, the cells were permeabilized with 0.1% Triton-X100 for 15 min and washed with PBS three times. Then cells were incubated with DAPI (Boster, China) for 5 min. The images were captured with fluorescence microscope (Evos fl auto, Life Technologies, United States) and images were taken at a magnification of 100-fold. BMSCs that undergo DNA replication during incubation present red fluorescence while the nucleus was represented with blue fluorescence.

### Cytoplasmic and Mitochondrial Protein Extraction

Cytoplasmic and Mitochondrial Protein Extraction Kit (Sangon Biotech, China) were used to separately extract cytoplasmic and mitochondrial protein according to manufacturer’s protocol. COX IV was used as internal reference of mitochondrial protein.

### Western Blot Analysis

Bone marrow mesenchymal stem cells were washed with PBS twice and covered with RIPA lysis buffer that containing 1% proteinase inhibitor cocktail for 30 min on ice. Protein concentration was determined by BCA method. Then 30 μg of each protein samples were loaded on 10% of sodium dodecyl sulfate-polyacrylamide gel electrophoresis (SDS-PAGE) and transferred to PVDF membranes (Millipore, Billerica, MA, United States). Then, the PVDF membranes were block by 5% bovine serum albumin for 1 h. The membranes were incubated overnight with specific primary antibodies at 4°C. In the next day, the membranes were incubated in 1:5000 diluted horseradish peroxidase-conjugated secondary antibody for 1 h (BA2913, Boster, China). Protein bands were visualized with Western ECL Substrate Kit (Thermo Pierce, United States). Images were recorded by Bio-Rad scanner (Hercules, CA, United States) and densitometry was analyzed by ImageJ version 1.48.

### Real-Time Polymerase Chain Reaction (RT-PCR)

Total mRNA expression was extracted with the total RNA extraction kit (Toyobo, Japan) according to the manufacturer’s instructions. Complementary DNA (cDNA) was synthesized from total RNA using first Strand cDNA Synthesis Kit (Toyobo, Japan). Then the cDNA was amplified with SYBR Green Real-time PCR Master Mix (Toyobo, Japan) with the following cycling conditions: 30 s of polymerase activation at 95°C, followed by 40 cycles of 95°C for 5 s and 60°C for 30 s. Glyceraldehyde-3-phosphate dehydrogenase (GAPDH) was selected as the internal control. Each cDNA sample was run in triplicates. Sequences of primers used were listed as follows: RUNX2: forward (CCCAGCCACCTTTACCTACA), reverse (ATGGAGTGCTGCTGCTGGTCTG); OPN: forward (GCGCTCTGTCTCTCTGACCT), reverse (ACCTTATTGCCCTCCTGCTT); GAPDH:forward (AACATCAAATGGGGTGAGGCC), reverse (GTTGTCATGGATGACCTTGGC).

### Statistical Analysis

All the data were represented as mean ± standard deviation (SD). Differences in numerical data between two groups were determined with Student’s two tailed *t*-test. One-way ANOVA was used to determine differences among groups more than two followed by a *post hoc* test. Statistical significance was defined as *p* < 0.05.

## Results

### Icariin Attenuated Iron Overload Induced Apoptosis of BMSCs

**FIGURE 1:**
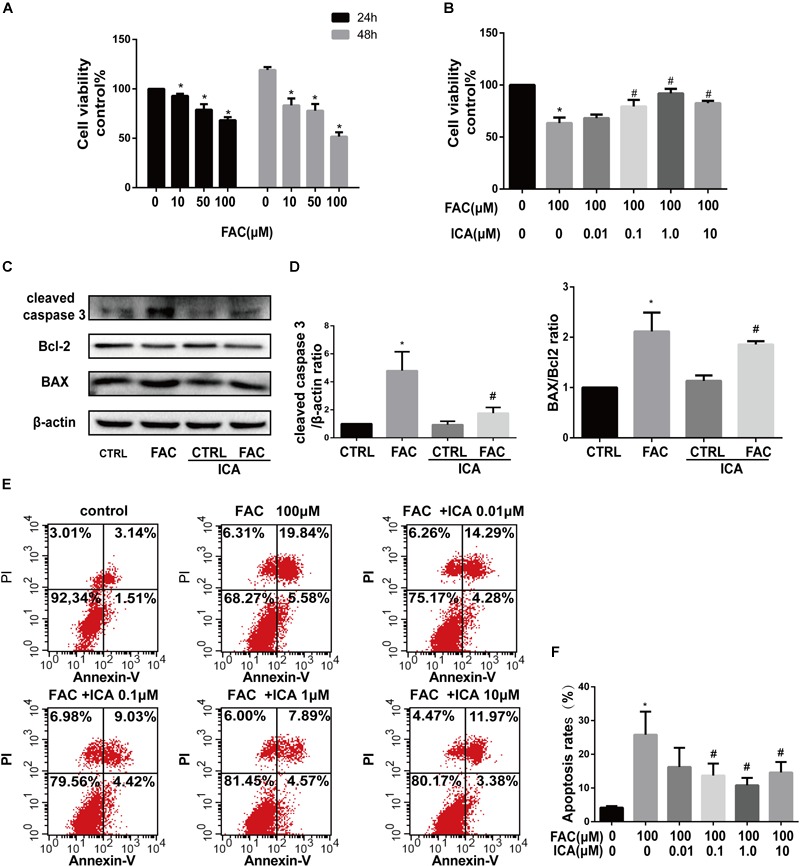
Icariin attenuated iron overload induced apoptosis of BMSCs. **(A)** The cytotoxicity of FAC on BMSCs viability was evaluated using the concentration of 0, 10, 50, and 100 μM after 24 and 48 h. ^∗^*P* < 0.05 versus control. **(B)** Icariin effectively attenuated the detrimental effects of FAC on BMSCs viability at concentrations of 0.1, 1, and 10 μM. ^∗^*P* < 0.05 versus control, ^#^*P* < 0.05 versus 100 μM FAC group. **(C)** Cleaved caspase-3, Bcl-2 and BAX protein levels were determined by Western blot analysis at 48 h. **(D)** Band density ratios of cleaved caspase-3 to β-actin and BAX to Bcl-2 in the Western blots were quantified by densitometry. ^∗^*P* < 0.05 versus control, ^#^*P* < 0.05 versus 100 μM FAC group. **(E)** Flow cytometric analysis of BMSCs stained with Annexin V-FITC/PI. **(F)** Percentage of apoptosis rates were expressed as means ± SD. ^∗^*P* < 0.05 versus control, ^#^*P* < 0.05 versus 100 μM FAC group.

Our results showed that FAC treatment decreased the viability of BMSCs in a dose dependent manner both after 24 and 48 h treatments (Figure 1A). FAC at concentrations of 10, 50, and 100 μM significantly decreased the viability of BMSCs with the most significant inhibitory effect at concentration of 100 μM (Figure 1A). However, icariin significantly attenuated the detrimental effects of FAC on BMSCs viability at concentrations of 0.1, 1, and 10 μM (Figure 1B). The protein expression of cleaved caspase-3, Bcl-2 and BAX was investigated by Western blot analysis. FAC (100 μM) treatment significantly increased cleaved caspase-3 protein expression. However, icariin (1 μM) treatment reversed the elevated cleaved caspase-3 expression induced by FAC (Figure 1C,D). Besides, 100 μM FAC treatments also significantly increased the BAX protein expression while decreased Bcl-2 protein expression. Icariin (1 μM) treatment significantly inhibited the FAC-induced increased in BAX/Bcl-2 ratio (Figure 1C,D). The protective roles of icariin on FAC induced BMSCs apoptosis was also investigated by Annexin V-FITC/PI double labeling with flow cytometric analysis. We found that 100 μM FAC dramatically increased the apoptosis of BMSCs compared to control groups (Figure 1E,F). Besides, icariin at concentrations of 0.1, 1, and 10 μM significantly inhibited the apoptotic effects of FAC on BMSCs (Figure 1E,F). We also found that 1 μM icariin has the most significant effects in preventing BMSCs from FAC induced apoptosis (Figure 1E,F). Taken together, the results revealed that icariin could significantly protect BMSCs from FAC overload induced apoptosis.

### Icariin Protected BMSCs Against Iron Overload Induced Collapse in Mitochondrial Membrane Potential (MMP)

**FIGURE 2:**
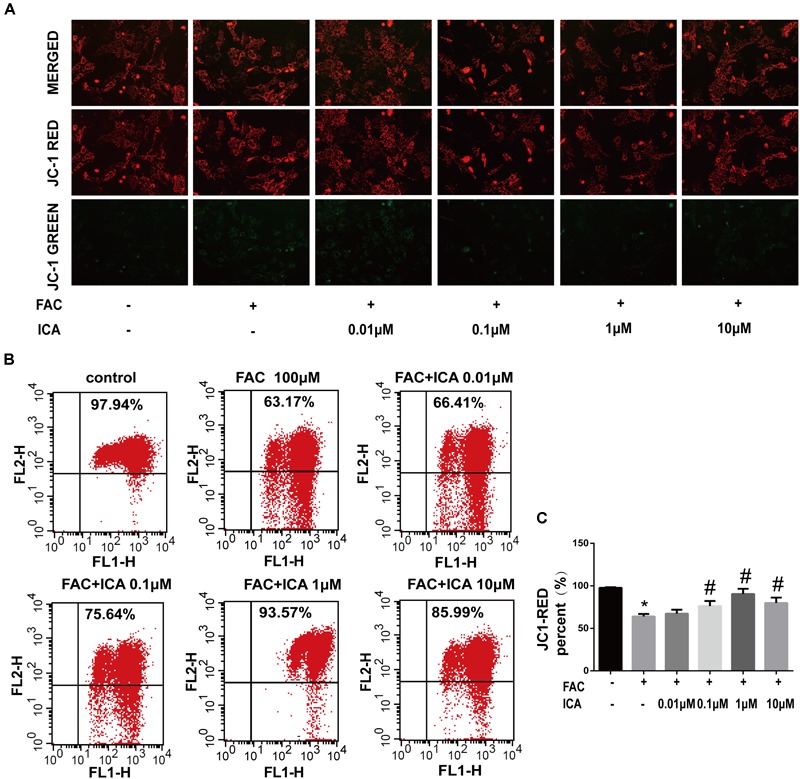
Icariin protected BMSCs against iron overload induced collapse in mitochondrial membrane potential (MMP). BMSCs were treated with 100 μM FAC or 100 μM FAC combined with icariin (0.01, 0.1, 1, and 10 μM) for 48 h. **(A)** Representative fluorescence images of MMP after incubating with JC-1. Red fluorescence represents JC-1 aggregates in healthy mitochondria whereas green fluorescence represents JC-1 monomer indicating MMP dissipation. Merged images represent co-localization of the JC-1 aggregates and JC-1 monomers. **(B)** Representative graphs of the flow cytometric analysis after incubating with JC-1. FL1 represents JC-1 green and FL2 represents JC-1 red. **(C)** Changes in the MMP were represented as the red fluorescence ratio and data were presented as means ± SD. ^∗^*P* < 0.05 versus control, ^#^*P* < 0.05 versus 100 μM FAC group.

Collapse in MMP represents mitochondrial dysfunction. To investigate whether icariin could protect BMSCs from FAC overload induced collapse in MMP, BMSCs were treated with 100 μM FAC or 100 μM FAC combined with icariin at concentrations from 0.01 to 10 μM. FAC (100 μM) treatment significantly increased the green fluorescence intensity of JC-1 monomers, suggesting that iron overload has a detrimental effect on the mitochondrial function leading to reduction of MMP (Figure 2A). However, we found that icariin treatment decreased the intensity of green fluorescence intensity of JC-1 monomers significantly suggesting that icariin has a protective effect against FAC induced mitochondrial dysfunction (Figure 2A). Flow cytometric analysis was also utilized to quantitatively measure the effects of FAC and icariin on MMP changes in BMSCs. It was shown that FAC (100 μM) treatment significantly inhibited the red fluorescence intensity of JC-1 aggregates, and icariin (0.1, 1, and 10 μM) treatment alleviated the inhibitory effects of FAC on JC-1 aggregates formation with the optimal concentration of 1 μM (Figure 2B,C). These results indicated that icariin protected BMSCs against iron overload induced collapse in MMP.

### Icariin Protected BMSCs Against Iron Overload Induced Reactive Oxygen Species (ROS) Generation

**FIGURE 3:**
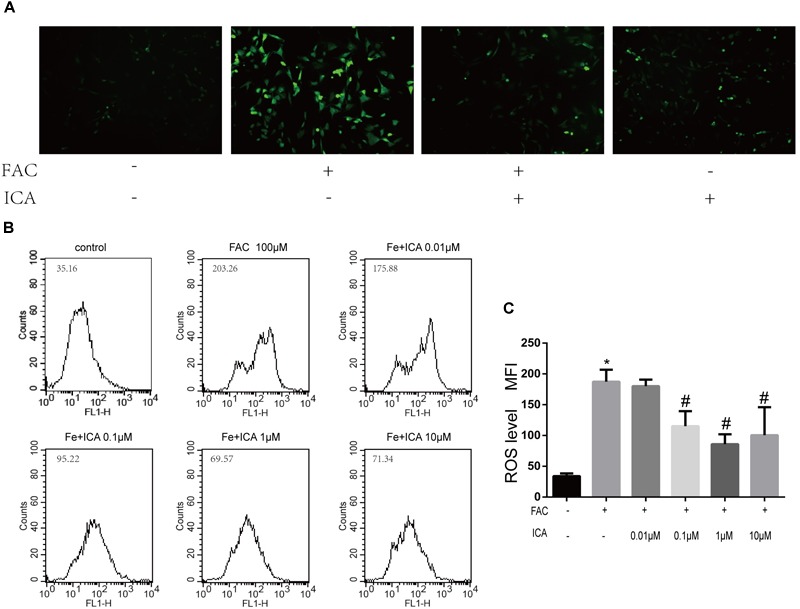
Icariin protected BMSCs against iron overload induced reactive oxygen species (ROS) generation. **(A)** Representative images of BMSCs with intracellular ROS stained by the fluorescence probe DCFH-DA after treatment with 100 μM FAC or 100 μM FAC combined with 1 μM icariin for 48 h. **(B)** Flow cytometric analysis of ROS production after staining with DCFH-DA. **(C)** Bar graphs showing the mean fluorescence intensity (MFI) of ROS levels in BMSCs. Data are shown as means ± SD. ^∗^*P* < 0.05 versus control, ^#^*P* < 0.05 versus 100 μM FAC group.

Previous studies have shown that iron overload could result in enhanced ROS formation in the mitochondria (Bresgen and Eckl, 2015). In the present study, we found that the intracellular ROS levels were maintained at low levels (Figure 3A). FAC (100 μM) treatment group showed a significant increase in green fluorescence intensity, representing increased ROS generation in the mitochondria (Figure 3A). We also found that icariin (1 μM) protected BMSCs against FAC overload induced mitochondrial ROS generation (Figure 3A). Flow cytometric analysis was also conducted to quantitatively investigate the effects of iron overload and icariin on ROS generation. We found that icariin inhibited FAC (100 μM) induced ROS generation in a dose dependent manner with the optimum concentration of 1 μM (Figure 3B,C).

### Icariin Protected BMSCs Against Iron Overload Induced Damage to the Mitochondria Through Modulating Mitochondrial Fusion and Fission

**FIGURE 4:**
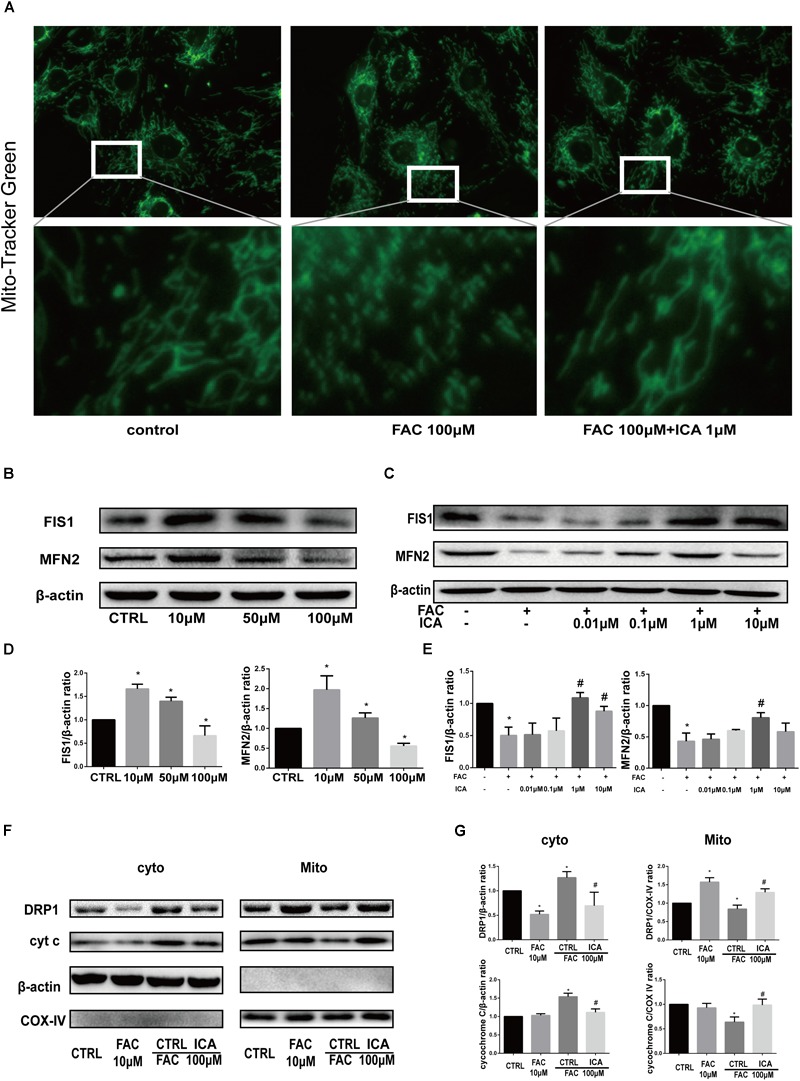
Icariin protected BMSCs against iron overload induced damage to the mitochondria through modulating mitochondrial fusion and fission. **(A)** Representative fluorescence images of the mitochondria (top row) and local amplification images of the selected area in the top row. Morphology of the mitochondria is stained with Mito-Tracker Green. The magnification time for the top row fluorescence images is 400. **(B)** BMSCs were treated with 10, 50, and 100 μM FAC for 48 h. The protein expression levels of FIS1 and MFN2 were evaluated with Western blot. **(C)** BMSCs were treated with 100 μM FAC and 100 μM FAC combined with different concentrations of icariin (0.01–10 μM) for 48 h. The protein expression levels of FIS1 and MFN2 were evaluated with Western blot. **(D)** The densitometric analysis of FIS1 and MFN2 protein expressions were normalized to β-actin. Data are shown as means ± SD. ^∗^*P* < 0.05 versus control. **(E)** The densitometric analysis of FIS1 and MFN2 protein expressions were normalized to β-actin. Data are shown as means ± SD. ^∗^*P* < 0.05 versus control, ^#^*P* < 0.05 versus 100 μM FAC group. **(F)** BMSCs were treated with 10, 100 μM FAC and 100 μM FAC combined with 1 μM icariin for 48 h. Cytosolic and mitochondrial proteins were separately extracted, relative expression of DRP1 and cytochrome C (cyt c) were evaluated with Western blot. COX IV was selected as the internal control for the mitochondrial protein. **(G)** The densitometric analysis of DRP1 and cyt c protein expressions was normalized to β-actin (cytosolic proteins) or COX IV (mitochondrial proteins). Data are shown as means ± SD. ^∗^*P* < 0.05 versus control, ^#^*P* < 0.05 versus 100 μM FAC group.

We found that the mitochondria in BMSCs of the control group showed a general wire-like shape. FAC (100 μM) treatment significantly increased the granulated mitochondria in BMSCs, while icariin (1 μM) restored the normal mitochondrial shape in BMSCs (Figure 4A). Besides, Western blot analysis was performed to investigate the protein expressions associated with mitochondrial fusion and fission. The expression of FIS1 and MFN2 was upregulated by 10 μM FAC and 50 μM FAC treatments at 48 h, but was downregulated by 100 μM FAC treatment at 48 h (Figure 4B,D). In addition, 1 and 10 μM icariin treatment for 48 h significantly abrogated the inhibitory effect of 100 μM FAC on FIS1 protein expression in BMSCs. The inhibitory effect of 100 μM FAC on MFN2 protein expression was also abrogated by 1 μM icariin treatment at 48 h (Figure 4C,E). We also found that 10 μM FAC induced DRP1 protein translocation from the cytoplasm to mitochondria at 48 h, while 100 μM FAC inhibited DRP1 protein translocation from the cytoplasm to mitochondria at 48 h, indicating that iron overload suppressed mitochondrial fission (Figure 4F,G). We also found that 10 μM FAC treatments for 48 h had no influence on cytochrome C protein expression both in the cytoplasm and mitochondria, while 100 μM FAC treatment also inhibited cytochrome C protein translocation from the cytoplasm to mitochondria compared to controls (Figure 4F,G). Interestingly, 1 μM icariin treatment reversed the effect of 100 μM FAC treatment on cytochrome C protein expression and translocation (Figure 4F,G). In summary, we found that iron overload significantly inhibited mitochondrial morphology and function, and 1 μM icariin could effectively maintain a normal mitochondrial morphology and function.

### Icariin Exerted Protective Effects on Iron Overload Induced Osteogenic Differentiation of BMSCs

**FIGURE 5:**
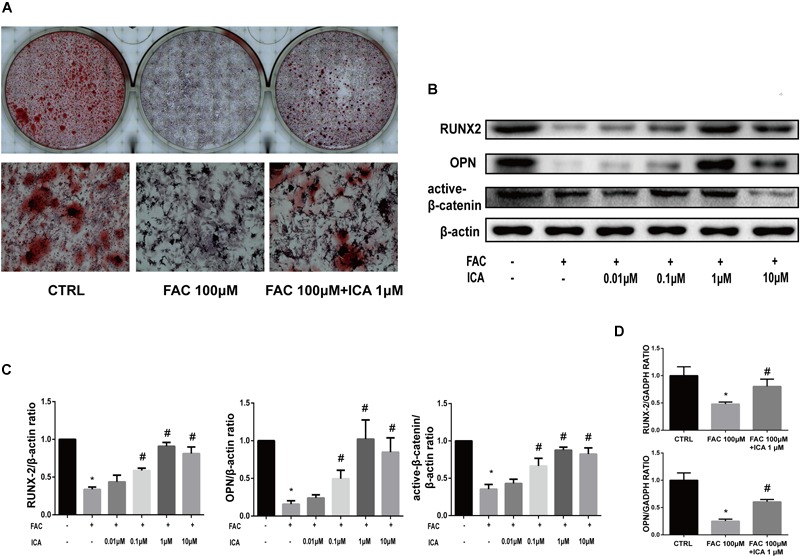
Icariin exerted protective effects on iron overload induced osteogenic differentiation of BMSCs. **(A)** BMSCs were cultured in osteogenic medium or osteogenic medium supplemented with 100 μM FAC or osteogenic medium supplemented with 100 μM FAC and 1 μM icariin. Alizarin Red staining was performed 21 days after cell culture to demonstrate extracellular mineralization. **(B)** BMSCs were cultured with osteogenic medium or osteogenic medium supplemented with 100 μM FAC or osteogenic medium supplemented with 100 μM FAC combined with different concentrations of icariin. RUNX2, OPN and active β-catenin protein expressions were investigated with Western blot after cell culture for 72 h. **(C)** The densitometric analysis of RUNX2, OPN and active β-catenin expressions were normalized to β-actin. Data are shown as means ± SD. ^∗^*P* < 0.05 versus control, ^#^*P* < 0.05 versus 100 μM FAC group. **(D)** BMSCs were cultured with osteogenic medium or osteogenic medium supplemented with 100 μM FAC or osteogenic medium supplemented with 100 μM FAC combined with 1 μM icariin. Relative expression levels of RUNX2 and OPN were evaluated by RT-PCR after 72 h. Data are shown as means ± SD. ^∗^*P* < 0.05 versus control, ^#^*P* < 0.05 versus 100 μM FAC group.

Previous investigations have shown that iron has both positive and negative effects on osteogenesis depending on its concentration (Katsumata et al., 2006; D’Amelio et al., 2008). In the present study, we tried to investigate whether iron overload impaired BMSCs osteogenic differentiation. BMSCs were cultured in osteogenic differentiation medium alone or supplemented with 100 μM FAC for 21 consecutive days. Alizarin Red staining was performed to investigate extracellular calcium depositions. We found that 100 μM FAC significantly inhibited the mineralization process of BMSCs (Figure 5A). To test whether icariin could protect against the inhibitory effect of iron overload on the osteogenesis of BMSCs, BMSCs were also cultured with osteogenic differentiation medium supplemented with 100 μM FAC and 1 μM icariin. We found that icariin significantly abolished the inhibitory effects of excessive iron ions on BMSCs osteogenic differentiation (Figure 5A). To further clarify the effect of iron overload and icariin on BMSCs osteogenic differentiation, we investigated the protein concentrations of Runx2, OPN and active β-catenin by Western blot. We found that 100 μM FAC significantly inhibited the expression of Runx2, OPN and active β-catenin compared to the control group (Figure 5B,C). Next, icariin with concentrations of 0.01–10 μM were added to the differentiation medium to investigate its effect on BMSCs osteogenic differentiation. We found that icariin (0.1, 1, and 10 μM) significantly promoted Runx2, OPN and active β-catenin protein expression with the optimal concentration of 1 μM (Figure 5B,C). The RT-PCR analysis also showed that 100 μM FAC dramatically inhibited Runx2 and OPN mRNA expression, while 1 μM icariin enhanced Runx2 and OPN mRNA expression (Figure 5D).

### Icariin Exerted Protective Effects on Iron Overload Induced Proliferation of BMSCs

**FIGURE 6:**
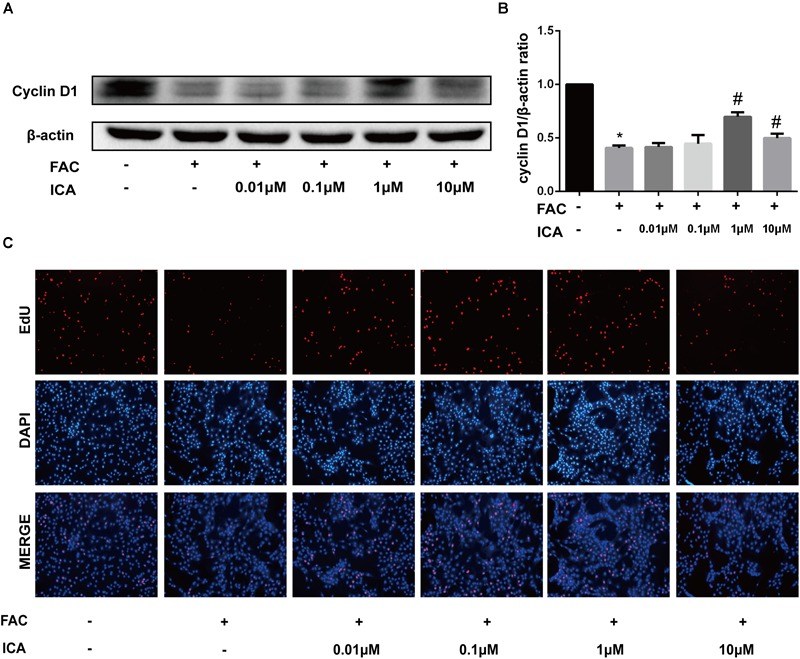
**(A)** BMSCs were treated with 100 μM FAC and 100 μM FAC combined with different concentrations of icariin (0.01–10 μM) for 48 h. Cyclin D1 protein expression was evaluated with Western blot. **(B)** The densitometric analysis of cyclin D1 protein expression was normalized to β-actin. Data are shown as means ± SD. ^∗^*P* < 0.05 versus control, ^#^*P* < 0.05 versus 100 μM FAC group. **(C)** Representative fluorescence images of Edu staining of BMSCs. Proliferating BMSCs positively stained with EdU showed red color. Cell nuclei stained with DAPI showed blue color.

The effect of iron overload on proliferation of BMSCs was investigated, we found that 100 μM FAC significantly inhibited the cyclin D1 protein expression (Figure 6A,B). Then, different concentrations of icariin were added to 100 μM FAC and Western blot analysis were used to determine the effect of icariin on iron overload induced BMSCs proliferation. It was shown that 1 and 10 μM icariin significantly increased the cyclin D1 protein expression at 48 h (Figure 6A,B). To further investigate the effect of iron overload and icariin on BMSCs proliferation, EdU staining was utilized. Results of the EdU staining showed that red fluorescence which represents proliferating BMSCs is significantly inhibited by 100 μM FAC treatment and promoted by icariin treatment (Figure 6C).

### Effect of Iron Overload and Icariin on the PI3K/AKT/mTOR Pathway and MAPK Pathway

**FIGURE 7:**
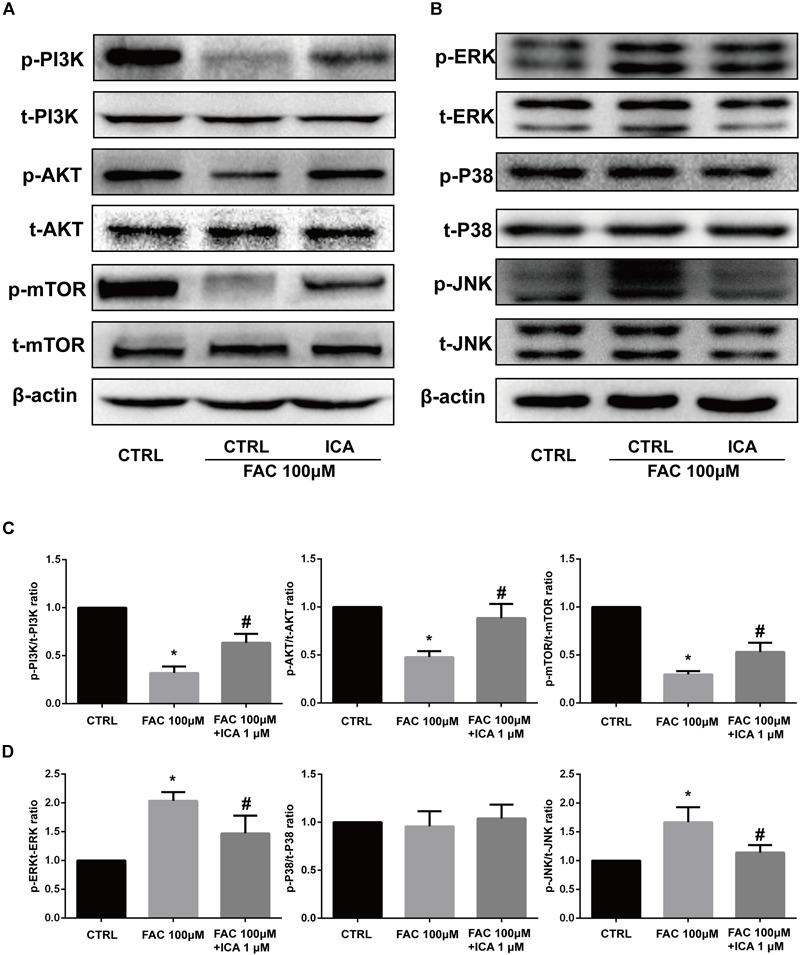
Effect of iron overload and icariin on the PI3K/AKT/mTOR pathway and MAPK pathway. BMSCs were treated with 100 μM FAC and 100 μM FAC combined with 1 μM icariin for 30 min. **(A)** Protein expression levels of t-PI3K, t-AKT, t-mTOR, p-PI3K, p-AKT, and p-mTOR were evaluated with Western blot. **(B)** Protein expression levels of t-ERK1/2, t-P38, t-JNK, p-ERK1/2, p-P38, and p-JNK were evaluated with Western blot. **(C)** The densitometric analysis of p-PI3K expression was normalized to t-PI3K, the densitometric analysis of p-AKT expression was normalized to t-AKT, and the densitometric analysis of p-mTOR expression was normalized to t-mTOR. Data are shown as means ± SD. ^∗^*P* < 0.05 versus control, ^#^*P* < 0.05 versus 100 μM FAC group. **(D)** The densitometric analysis of p-ERK expression was normalized to t-ERK, the densitometric analysis of p-P38 expression was normalized to t-P38, and the densitometric analysis of p-JNK expression was normalized to t-JNK. Data are shown as means ± SD. ^∗^*P* < 0.05 versus control, ^#^*P* < 0.05 versus 100 μM FAC group.

The phosphatidylinositol 3-kinase (PI3K)–AkT–mammalian target of rapamycin (mTOR) signaling cascade is a key regulator of cellular functions and balances between cell survival and apoptosis (Ma et al., 2018). We found that 100 μM FAC treatments significantly inhibited the phosphorylation of PI3K, AkT, and mTOR at 30 min. We also found that 1 μM icariin significantly abrogated the inhibitory effects of iron overload on the PI3K/AKT/mTOR pathway, leading to increased phosphorylation of PI3K, AkT, and mTOR at 30 min (Figure 7A,C). Besides, the effects of iron overload and icariin on the mitogen-activated protein kinase (MAPK) pathway was also investigated. MAPK superfamily includes the extracellular signal-regulated protein kinase 1/2(ERK1/2), p38 MAPK (p38) submodules and C-Jun N-terminal kinase (JNK) (Widmann et al., 1999). We found that 100 μM FAC treatments significantly increased the phosphorylation of ERK1/2 and JNK, but did not affect the phosphorylation of p38 (Figure 7B,D). Besides, 1 μM icariin significantly inhibited the phosphorylation of ERK1/2 and JNK induced by iron overload (Figure 7B,D). These results demonstrated that iron overload suppressed the PI3K/AKT/mTOR pathway and activated the ERK1/2 and JNK pathways. Icariin reversed the effects of iron overload via activating the PI3K/AKT/mTOR pathway and inhibiting the ERK1/2 and JNK pathways.

## Discussion

Recently, more and more investigations showed that iron overload in circumstances such as hereditary hemochromatosis or multiple blood transfusions in patients with β-thalassaemia exerted toxic effects on the proliferation and differentiation of BMSCs (Suzuki et al., 2008; Danjou et al., 2013). Moreover, iron overload is also directly related to bone mass loss and osteoporosis (Li et al., 2012). However, it still remains elusive how to effectively prevent iron overload induced injury to the BMSCs.

Our previous studies have shown that icariin could significantly promote the proliferation and osteogenic differentiation of ASCs (Ye et al., 2017). In the present study, we found that icariin with concentrations from 0.1 to 10 μM could significantly attenuate the detrimental effects of 100 μM FAC on BMSCs viability. BAX exerts pro-apoptotic function by inducing cytochrome C (cyt c) translocation from the mitochondria to cytosol and finally leads to activation of cleaved caspase-3 and cell apoptosis (Liman et al., 2013). Bcl-2 is anti-apoptotic protein that modulates the execution of cell death pathway (Cory and Adams, 2002). In this study, we also found that 1 μM icariin significantly suppressed the expression of cleaved caspase-3 and BAX induced by 100 μM FAC and promoted Bcl-2 expression. The flow cytometric results further demonstrated that 1 μM icariin significantly suppressed 100 μM FAC induced apoptosis of BMSCs. Similarly, Jiao et al. (2018) also demonstrated that icariin could significantly increase the migration capacity of rabbit BMSCs with the optimal concentration of 1 μM.

Iron overload in cells could result in highly reactive free radicals (HO^∙^ and RO^∙^) by interacting with H_2_O_2_ and finally result in increased ROS generation (Dixon and Stockwell, 2014). Excessive ROS generation impaired the structure and functions of mitochondria, promoted cleaved caspase-3 expression and activated the mitochondrial apoptotic pathway. Previous studies have shown that icariin could significantly decrease intracellular ROS levels in podocytes induced by high glucose (Qiao et al., 2018). In this study, we found that 100 μM FAC significantly induced mitochondrial ROS generation, collapse of the MMP and destruction of the mitochondrial structure, whose effects were notably attenuated by icariin treatment. FIS1 is mitochondrial fission protein and MFN2 is mitochondrial fusion protein (Reddy et al., 2011). In this study, we also found that the expression of mitochondrial fission protein FIS1 and fusion protein MFN2 were promoted by 10 and 50 μM FAC treatments while inhibited by 100 μM FAC treatments. Moreover, 1 μM icariin significantly attenuated the inhibitory effects of 100 μM FAC on BMSCs leading to increased expression of FIS1 and MFN2. Previous investigations have also revealed that mitochondrial stress could induce more DRP1 migration from the cytoplasm to mitochondria, and DRP1 could regulate the mitochondrial fission by migrating to and locating on the mitochondrial surface (Wang and Song, 2018). Besides, extensive mitochondrial stress is also closely related to cell apoptosis caused by liberation of the pro-apoptotic factor such as cyt c from mitochondria to the cytoplasm (Zhou et al., 2017). In the present study, we found that 10 μM FAC significantly promoted DRP1 and cyt c translocation from the cytoplasm to mitochondria while 100 μM FAC significantly inhibited DRP1 and cyt c translocation from the cytoplasm to mitochondria. Icariin attenuated the inhibitory effects of 100 μM FAC on DRP1 and cyt c translocation. These results indicated that icariin could potentially attenuate iron overload induced oxidative injury to BMSCs by regulating both mitochondrial fusion and fission.

Our results also revealed that 100 μM FAC treatment inhibited the osteogenic differentiation of BMSCs *in vitro*. FAC treatment significantly inhibited RUNX2, OPN and active β-catenin protein expression. In addition, icariin with concentrations from 0.1 to 10 μM significantly attenuated the inhibitory effects of 100 μM FAC on BMSCs osteogenic differentiation. Besides, our RT-PCR also showed that expression of the osteoblast-specific genes (RUNX2 and OPN) was also inhibited by FAC treatment, which effect was remarkably attenuated by 1 μM icariin treatment. To investigate whether 100 μM FAC could inhibit the proliferation of BMSCs, cyclin D1 protein expression was analyzed. We found that 100 μM FAC treatments inhibited the proliferation of BMSCs, which was reversed by icariin supplementation with concentrations of 1 and 10 μM. Consistently, EdU immunofluorescence staining also demonstrated that BMSCs proliferation was inhibited when treated with 100 μM FAC and was promoted when treated with 100 μM FAC and 1 μM icariin.

The PI3K–AKT–mTOR signaling pathway is critical for several cellular functions and could balance between cell survival and apoptosis (Wang S. et al., 2018). Iron overload significantly activated the PI3K/AKT pathway in hippocampal neurons and caused neurotoxicity (Uranga et al., 2013). However, iron overload was shown to inhibit the PI3K/AKT pathway in preosteoblast cells leading to cell arrest and autophagy (Cen et al., 2018). In the present study, we found that 100 μM FAC treatments significantly inhibited the phosphorylation of PI3K, AKT, and mTOR, which effects were attenuated by 1 μM icariin treatment. The MAPK superfamily is also involved in the proliferation, differentiation and stress reaction of cells (Widmann et al., 1999). MAPK superfamily includes the ERK1/2, p38 submodules, and JNK (Widmann et al., 1999). In the present study, we found that 100 μM FAC treatments significantly increased the phosphorylation of ERK1/2 and JNK, but expression of p-P38 was not affected. Icariin attenuated the effect of FAC on phosphorylation of both ERK1/2 and JNK, but did not affect the expression of p-P38.

In summary, iron overload impaired the normal structure and function of the mitochondrial in BMSCs by increasing ROS generation and inducing collapse of the MMP. In addition, iron overload inhibited the proliferation and osteogenic differentiation of BMSCs. Moreover, iron overload suppressed the PI3K/AKT/mTOR signaling pathway and activated both ERK1/2 and JNK signaling pathways. Icariin could effectively attenuate the detrimental effects caused by iron overload in BMSCs potentially by regulating mitochondrial fusion and fission, PI3K/AKT/mTOR and MAPK pathways. This study has illustrated the molecular mechanisms of the dysfunction of BMSCs caused by iron overload and provided a novel idea for the utility of icariin in clinical problems such as bone deficiency caused by iron overload.

## Author Contributions

XY, YY, and FG conceived and designed the experiments. XY, XJ, KS, YD, and YZ performed the experiments. JG and FG analyzed the data. JG and YZ contributed reagents, materials, and analysis tools. JG and YY wrote the manuscript.

## Conflict of Interest Statement

The authors declare that the research was conducted in the absence of any commercial or financial relationships that could be construed as a potential conflict of interest.

## Supplementary Material

FIGURE S1 Chemical structure for icariin.
