# Supplementary figures and images for: Icariin Protects Bone Marrow Mesenchymal Stem Cells Against Iron Overload Induced Dysfunction Through Mitochondrial Fusion and Fission, PI3K/AKT/mTOR and MAPK Pathways
Source: Front Pharmacol. 2019 Feb 28;10:163. doi: 10.3389/fphar.2019.00163 (PMC6403125; doi:10.3389/fphar.2019.00163)

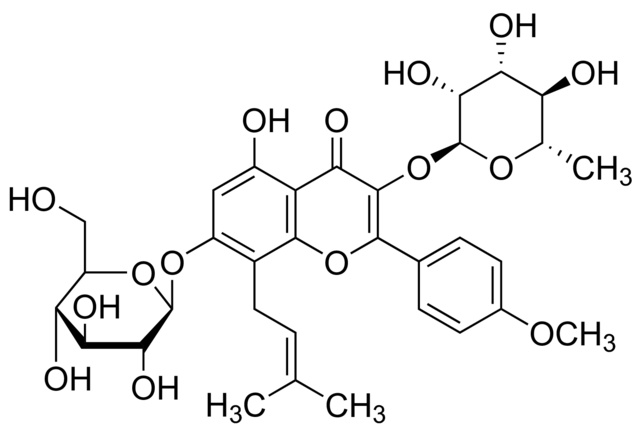

Supplement: FIGURE S1 — Chemical structure for icariin. [file Image_1.PNG]
